# Intraspecific variation of morphological traits backed up with molecular evidence votes for re-appraisal of hitherto distinguished *Balaustium* species—a case study of *Balaustium murorum* (Acariformes: Parasitengona, Erythraeidae)

**DOI:** 10.1007/s10493-023-00859-3

**Published:** 2023-11-02

**Authors:** Joanna Mąkol, Magdalena Felska

**Affiliations:** Department of Invertebrate Systematics and Ecology, Institute of Environmental Biology, Wrocław University of Environmental and Life Sciences, Kożuchowska Str. 5b, 51-631 Wrocław, Poland

**Keywords:** *Balaustium* spp., Metric data, Leg chaetotaxy, COI gene, Intraspecific variation

## Abstract

Molecular examination of representatives of *Balaustium* from several populations in SW Poland, performed using the sequence data from the mitochondrial cytochrome c oxidase subunit I, confirmed their common specific affiliation and identity with *Balaustium murorum*. The potential presence of distinct species in the studied material, preliminarily inferred from the discovery of clusters as a result of Principal Component Analysis exploring the metric data sets, was rejected due to the finding of only one haplotype, at intra- and inter-population sampling. An insight into meristic traits in larvae, focused on chaetotaxy of legs, revealed wider variation than hitherto recognized for the species. The variation was higher in laboratory-reared larvae compared to field-collected ones. The overall deviations from the mean character values at intra- and interpopulation levels, higher than hitherto observed for the species, vote for the reappraisal of the criteria adopted for discrimination of members of *Balaustium* with the application of an integrative approach.

## Introduction

The genus *Balaustium* von Heyden, which counts 42 valid species described to date (Mąkol and Wohltmann 2012, 2013; Kamran and Alatawi 2014; Noei et al. 2017, 2019; Šundić and Noei 2021), has 27 species distributed in the Palearctic. Of those, 14 species have been known exclusively from larvae, 10 from active postlarval forms (adults and/or deutonymphs), and only three from both.

The members of *Balaustium* have been known for their mass appearance in sunny places, on flowering vegetation but also on stone or concrete surfaces. Larval parasitism, contrary to most other Parasitengona mites, has not been confirmed and at least for some species the larvae are pollinivorous or feed on eggs of some invertebrates (e.g., Putman 1970; Childers and Rock 1981; Mąkol et al. 2012). Some *Balaustium* spp. have been reported as potential biocontrol agents, but also as agricultural pests and mites attacking humans (Muñoz-Cárdenas et al. 2015, and references therein). The identification of *Balaustium* species has been based on morphology, at poorly indicated species boundaries, and unrecognized intraspecific variation caused by usually small sample sizes.

*Balaustium murorum* (Hermann), considered the most common member of the genus, has been known to be widely distributed in the Western Palearctic; however, according to recent data (Shimano and Hiruta 2022) it is present also in Japan. For several decades the species has been known exclusively from active postlarval forms. The lack of males in the analyzed material supported the hypothesis of asexual reproduction in *B. murorum* (e.g., Witte 1975). The redescription of active postlarval forms combined with first description of laboratory-reared larvae obtained from field-collected females, was provided by Mąkol (2010). The eggs of *B. murorum* are known to constitute the hibernating instar (Wohltmann 2000).

Here, we aim to test the hypothesis about wider (than hitherto recognized) variation of morphological traits in larvae of *B. murorum*, observed on increased sample size. We infer intraspecific variation based on an integrative approach, employing both molecular and morphological traits. The alternative hypothesis assumes the presence of distinct species, differing with respect to measurable traits, in the material originating from different localities/populations. The results of our studies may shed light on future assessments of the usefulness of selected morphological traits in species diagnosis and discrimination.

## Materials and methods

### Sampling

The study material comprised representatives of *Balaustium* collected at various localities in SW Poland: (I) Wrocław Swojczyce (51°06′10″N, 17°07′53″E, 117 m above sea level, concrete plate at the side of cultivated field, 16 June 2021, leg. JM [coll. no. 8250]), (II) Wrocław Swojczyce (51°06′30″N, 17°08′01″E, 118 m a.s.l., concrete platform and stairs between cycling/walking path and cultivated field, 1 June 2022, leg. JM [coll. no. 8412]), (III) Wrocław Ołbin (51°07′31″N, 17°03′23″E, 120 m a.s.l., brick wall balcony frame, 29 April 2014, leg. JM [coll. no. WW29/B2]), (IV) Miłosławice (51°30′13″N, 17°11′32″E, 113 m a.s.l., bricks and *Pelargonium* sp., 4 July 2021, leg. A. Derdak [coll. no. 8251 and 8252]), (V) Bystrzyckie Mts, Niemojów (50°09′36″N, 16°34′21″E, 553 m a.s.l., concrete-coated stone wall, partly covered with moss, 18 May 2013, leg. M. Konikiewicz [coll. no. 5728]). Monitoring of natural populations from which material was collected for the purpose of morphological and molecular analyses was carried out to trace the periodic occurrence of instars during the growing season.

### Rearing

The field-collected females (localities I, IV) were placed separately in the rearing vials filled with charcoaled Plaster of Paris. The material was kept in a MLR-352 H climate chamber [L12(22 °C):D12(15 °C) h photoperiod] until oviposition and for 13–16 following weeks, then in the fridge (4 °C) for ca. 7 weeks, followed by ambient room temperature until the emergence of larvae. Females were transferred to EtOH after oviposition. Larvae, not supplied with food, were transferred to EtOH up to a few days after emergence.

### DNA extraction, PCR amplification and sequencing, and molecular analysis

Field-collected representatives of active postlarval forms (localities I–III), larvae obtained by experimental rearing and field-collected postlarval forms (locality IV), as well as field-collected larvae (locality V), randomly selected from each of five populations served for molecular analyses. A non-destructive method of DNA extraction was applied, aimed at retaining the exoskeletons for morphological examination. Total genomic DNA was extracted using a DNeasy Blood and Tissue Kit (Qiagen, Hilden, Germany). In DNA extraction, isolation, and amplification we followed Trnka et al. (2023). Amplification of the cytochrome c oxidase subunit 1 (COI) fragment was carried out using two pairs of primers covering the same region of the COI gene: degenerate forward primer bcdF01 (5′-CATTTTCHACTAAYCATAARGATATTGG-3′) and reverse primer bcdR04 (5′-TATAAACYTCDGGATGNCCAAAAAA-3′) (Dabert et al. 2010) or forward primer Bala_COIF (5′-TTTAGGAGTATGATCAGGTATTTTTGG-3′) and reverse primer Bala_COIR (5′-GTTGATATAAGATTGGGTCTCCTCCTC-3′) (Hiruta et al. 2018).

The amplicons were sequenced in both directions (Genomed, Warsaw, Poland). The single haplotype sequence is deposited in the GenBank under accession number OR644014. For comparison, the following COI sequences of *Balaustium* spp. (Hiruta et al. 2018) were retrieved from GenBank: three sequences of *Balaustium murorum* (acc. nos. LC260367.1, LC260351.1, LC260374.1), one of *Balaustium* sp. 1 (LC260370.1), one of *Balaustium* sp. [?]1 (LC260361.1), two of *Balaustium* sp. 2 (LC260343, LC260344.1), and one of *Balaustium* sp. 3 (LC260379.1). The multiple sequence alignment, followed by the calculation of distances between sequences (default parameters), was performed in Geneious Prime (https://www.geneious.com).

### Morphological examination

Thirty laboratory-reared larvae, obtained from field-collected females (localities I, IV) as well as 16 larvae collected directly in the field (locality V), including the exoskeletons recovered after DNA extraction, were subject to morphological analyses. The material preserved in EtOH was mounted on microscopic slides in Hoyer’s medium. The analyses were carried out in Nikon Eclipse E-600 / 80i compound microscopes, equipped with differential interference contrast and DS-Fi1 / DS-Fi3 camera systems, using NIS-Elements D software (https://www.microscope.healthcare.nikon.com/products/software/nis-elements/nis-elementsdocumentation).

The meristic traits (chaetotaxy of selected pedipalp and leg segments) were analyzed, commonly used in the diagnosis and identification of *Balaustium* spp. based on larvae. The chaetotaxy was assessed for each larva on both sides of the symmetry axis. In ascertaining the number of setae only the unambiguous states were considered (segments that were damaged or superimposed with other parts of the body were excluded from analyses). The terminology and abbreviations follow Mąkol (2010). The standard measurements, given in micrometers, are presented as a range, followed by sample size, mean value, and coefficient of variation.

### Statistical analyses

All analyses were performed using R statistical software (R Core Team 2023). As the normal distribution was not confirmed, non-parametric ANOVA (Kruskal-Wallis) was used to test morphological variation between populations (I, IV: larvae obtained by experimental rearing of field-collected females; V: larvae collected in the field). We performed a statistical procedure with the null hypothesis stating that distributions of random vectors are identical for all considered groups (populations I, IV, V). This null hypothesis was rejected (α = 0.05). The Kruskal-Wallis tests were applied separately for all dependent variables and the false discovery rate was controlled using the Benjamini-Hochberg stepwise adjustment. The Kruskal-Wallis test was followed by Dunn’s multiple comparison for each statistically significant variable.

To discover clusters based on the similarity between objects, the Principal Component Analysis (PCA) was applied, which is commonly used for dimensionality reduction and classification. The metric traits that are commonly applied in diagnosis of the members of *Balaustium* spp. and, at the same time, those that showed significant differences between the three studied populations, were considered in the analysis (for the finally selected set of variables see the Results section). Only larvae with the complete set of measurements were included in the PCA. The principal components were visualized using ‘ggbiplot’ v.0.55 function of the biplot package in R.

### Reference material storing

The slide-mounted material, including exoskeletons that remained after DNA extraction, is deposited at the Department of Invertebrate Systematics and Ecology, Wrocław University of Environmental and Life Sciences.

## Results

### Observations on development and phenology

The oval, dark brown eggs were laid by 15 females out of 38 individuals intended for the laboratory rearing. The transition to the prelarval stage, expressed in the rupture of the chorion and the appearance of a lighter stripe (deutoval sheath) in the equatorial plane, occurred after the eggs were transferred from 4 ºC to ambient temperature. In natural populations, active development stages were observed from April to the turn of June/July. Larvae were the first to appear in the spring (April-May), whereas the appearance of deutonymphs and then adults fell in April-June. Starting from the beginning of July at the latest, the total absence of the active instars was recorded.

### Molecular identification

We obtained seven COI sequences from specimens originating from all five localities. As a result of alignment and cutting we received a compact 550 bp data block. All examined specimens shared the same haplotype. They revealed 99.8–100% identity with sequences of *B. murorum* retrieved from the GenBank and obtained by Hiruta et al. (2018) from specimens collected in Graz (Austria) (100%), Tokyo (Japan) (100%), and Fukuoka (Japan) (99.8%). The distance between *B. murorum* (including our specimens and the above-mentioned specimens from Austria and Japan) and other non-*murorum* members of *Balaustium* collected from various localities in Japan (Hiruta et al. 2018) ranged between 10.5 and 18.2%.

### Morphological identification

The standard measurements given separately for larvae obtained by experimental rearing from field-collected females (localities I and IV) and for field-born larvae (locality V) were compiled in Table 1. The extent of variability in relation to the mean of the population (coefficient of variation, CV) was higher in the case of field-born larvae compared to laboratory-reared ones (32 out of 45 characters revealed a higher value of CV).

**Table 1:** Morphometric data on larvae of *Balaustium murorum*

Character	After Mąkol 2010 (n = 3)	Present study (localities I and IV)^a^ (laboratory-reared larvae)	Present study (locality V)^b^ (field-born larvae)	Data aggregated (D = A + B + C)
	min–max	min–max (sample size, mean, CV)	min–max (sample size, mean, CV)	min–max
	A	B	C	D
LB	367–426	309–416 (30, 373, 6.76)	349–683 (16, 566, 16.85)	309–683
WB	235–250	183–252 (30, 217, 7.10)	249–459 (16, 363, 17.74)	183–459
PaTr	20–21	16–22 (30, 19, 8.34)	17–22 (15, 19, 9.10)	16–22
PaFe	50–54	42–52 (30, 48, 4.82)	45–55 (15, 49, 6.07)	42–55
PaGe	31–35	28–36 (30, 32, 5.48)	30–39 (15, 35, 7.32)	28–39
PaTi	13–14	12–15 (30, 14, 5.10)	12–16 (15, 14, 6.90)	12–16
PaTa	18–19	16–21 (30, 18, 7.24)	15–20 (15, 17, 9.79)	15–21
Odo	21–22	18–22 (30, 21, 5.07)	16–20 (15, 18, 8.31)	16–22
ASens	38–43	36–43 (29, 39, 4.24)	33–44 (15, 37, 7.30)	33–44
SBa	12–13	12–21 (30, 15, 17.73)	11–14 (16, 13, 7.32)	11–21
PSens	55–60	52–61 (30, 56, 5.22)	48–64 (15, 56, 8.72)	48–64
SBp	12–15	11–16 (30, 14, 10.58)	12–14 (15, 13, 7.07)	11–16
ISD	48–54	46–59 (30, 53, 6.40)	55–65 (16, 58, 5.25)	46–65
DS	24–35	29–34 (30, 32, 4.49)	27–37 (16, 32, 7.36)	24–37
1a (St I)	44–46	35–44 (30, 41, 6.87)	37–49 (14, 44, 7.80)	35–49
2a (St II)	36–39	30–40 (30, 34, 7.83)	30–39 (14, 32, 8.16)	30–40
Co I	43–46	40–49 (30, 43, 5.57)	36–42 (14, 39, 3.51)	36–49
Co II	38–44	34–42 (30, 39, 5.67)	32–40 (14, 36, 6.82)	32–44
Co III	32–37	30–41 (30, 36, 8.38)	30–39 (14, 35, 7.10)	30–41
Cx I	55–60	47–59 (30, 53, 6.91)	48–63 (16, 56, 6.79)	47–63
Tr I	29–32	23–33 (30, 28, 8.46)	24–36 (16, 29, 9.62)	23–36
bFe I	43–50	39–49 (30, 44, 6.17)	36–47 (16, 43, 7.85)	36–50
tFe I	41–48	38–48 (30, 44, 5.68)	39–49 (16, 44, 6.73)	38–49
Ge I	73–80	68–82 (30, 75, 5.16)	67–81 (16, 76, 5.19)	67–82
Ti I	72–83	67–79 (30, 74, 4.09)	69–82 (16, 76, 4.89)	67–83
Ta I (L)	65–73	57–67 (30, 62, 4.35)	53–65 (16, 60, 5.22)	53–73
Ta I (W)	29–30	23–30 (30, 27, 6.72)	24–34 (16, 28, 10.60)	23–34
Leg I	380–417	383–431 (30, 406, 3.22)	368–437 (16, 408, 4.78)	368–437
Cx II	56–65	49–61 (30, 56, 5.42)	48–67 (16, 58, 7.62)	48–67
Tr II	25–31	23–30 (30, 26, 5.76)	22–33 (16, 28, 12.53)	22–33
bFe II	36–42	32–38 (30, 35, 5.76)	30–43 (16, 36, 9.59)	30–43
tFe II	35–40	31–40 (30, 36, 5.63)	34–41 (16, 37, 4.75)	31–41
Ge II	61–68	52–67 (30, 60, 6.47)	53–67 (16, 62, 5.65)	52–68
Ti II	60–65	53–64 (30, 61, 5.07)	59–66 (16, 63, 3.63)	53–66
Ta II	58–66	46–56 (30, 52, 4.41)	47–58 (16, 51, 5.33)	46–66
Leg II	335–370	304–341 (30, 327, 3.32)	298–349 (16, 335, 3.98)	298–370
Cx III	61–64	54–64 (30, 58, 4.91)	54–63 (16, 58, 4.04)	54–64
Tr III	31–35	21–28 (30, 26, 6.97)	25–33 (16, 29, 8.74)	21–35
bFe III	32–41	33–42 (30, 37, 5.74)	32–44 (16, 38, 9.59)	32–44
tFe III	43–49	41–48 (30, 44, 4.04)	38–49 (16, 43, 10.14)	38–49
Ge III	66–70	52–72 (30, 65, 6.46)	63–73 (16, 69, 5.18)	52–73
Ti III	76–80	65–80 (30, 74, 6.23)	69–83 (16, 76, 6.01)	65–83
Ta III	60–64	49–60 (30, 54, 5.35)	47–56 (16, 52, 5.30)	47–64
Leg III	381–390	335–382 (30, 358, 3.81)	342–389 (16, 366, 3.52)	335–390
IP	1098–1169	1031–1152 (30, 1091, 3.10)	1037–1167 (16, 1109, 3.31)	1031–1169

^a^Unengorged specimens

^b^Specimens at different phase of engorgement

Contrary to the metric data, the number of setae arising at palpfemur, palpgenu and on each segment of legs I-III revealed higher variation in laboratory-reared compared to field-born larvae (Table 2). The frequency of occurrence of states that departed from most common one observed for given character (here assumed as present in at least 66% of individuals) was higher in laboratory-reared larvae. In no case, except for tibia I, II, III and genu II, III the states departing from the most observed for a given character were confirmed on both sides of the symmetry axis.

**Table 2:** Chaetotaxy of selected pedipalp segments and of leg segments in larvae of *Balaustium murorum* examined in the present survey

Character	Character state variants^a^	Localities I and IV (laboratory-reared larvae)		Locality V (field-born larvae)	
		Frequency of occurrence (%)^b^	Share (%) of specimens with an equal state confirmed on both sides of the symmetry axis^c, d^	Frequency of occurrence (%)^b^	Share (%) of specimens with an equal state confirmed on both sides of the symmetry axis^c, d^
PaFe	1n	99	97	100	100
	0n	1	0	–	–
PaGe	2n	95	89	96	93
	3n	3	0	4	0
	1n	2	0	–	–
Cx I	1n	100	100	100	100
Tr I	3n	100	100	100	100
bFe I	4n	89	71	100	100
	5n	9	0	–	–
	3n	2	0	–	–
tFe I	5n	94	87	96	90
	6n	2	0	4	0
	4n	2	0	–	–
	3n	2	0	–	–
Ge I	8n, 1σ, 1κ	87	77	93	86
	8n, 2σ, 1κ	–	–	7	0
	7n, 1σ, 1κ	4	0	–	–
	6n, 1σ, 1κ	4	0	–	–
	9n, 1σ, 1κ	2	0	–	–
	3n, 0σ, 1κ	2	0	–	–
	7n, 0σ, 0κ	2	0	–	–
Ti I	11n, 2ϕ, 1κ	37	37	54	50
	11n, 3ϕ, 1κ	37	19	42	42
	10n, 3ϕ, 1κ	15	6	4	0
	12n, 2ϕ, 1κ	4	0	–	–
	12n, 3ϕ, 1κ	2	0	–	–
	11n, 2ϕ, 2κ	2	0	–	–
	11n, 1ϕ, 1κ	2	0	–	–
Ta I^e^	20–24n^f^, 1ω, 2ζ, 1z	100	55	93	60
	19n, 2ω, 2ζ, 1z	–	–	7	0
Cx II	1n	100	100	100	100
Tr II	3n	98	96	100	100
	2n	2	0	–	–
bFe II	4n	94	87	100	100
	5n	6	0	–	–
tFe II	5n	89	77	100	100
	4n	7	0	–	–
	6n	4	0	–	–
Ge II	8n, 1κ	71	61	100	100
	8n, 1σ, 1κ	15	9	–	–
	7n, 1κ	8	4	–	–
	8n	4	0	–	–
	7n	2	0	–	–
Ti II	11n, 2ϕ	53	17	78	60
	10n, 2ϕ	21	6	–	–
	9n, 2ϕ	6	6	–	–
	10n, 3ϕ	6	6	–	–
	11n, 3ϕ	6	0	22	20
	12n, 2ϕ	2	0	–	–
	11n, 2ϕ, 1κ	2	0	–	–
	11n, 1ϕ, 1κ	2	0	–	–
Ta II^e^	(15)17–22n, 1ω, 2ζ, 1z	100	33	–	–
	18–20n, 1ω, 2ζ, 1z	–	–	100	50
Cx III	1n	98	97	100	100
	0n	2	0	–	–
Tr III	2n	100	100	100	100
bFe III	2n	87	70	100	100
	3n	12	0	–	–
	1n	2	0	–	–
tFe III	5n	96	100	96	91
	4n	4	0	4	0
Ge III	8n	89	87	100	100
	9n	6	4	–	–
	7n	6	0	–	–
Ti III	11n, 1ϕ	66	42	100	100
	10n, 1ϕ	26	8	–	–
	13n, 1ϕ	2	0	–	–
	12n, 1ϕ	2	0	–	–
	9n, 1ϕ	2	0	–	–
	11n, 2ϕ	2	0	–	–
Ta III^e^	18–21n, 1ζ	100	58	–	–
	19–21n, 1ζ	–	–	95	56
	19–21n, 2ζ	–	–	5	0

^a^Character states on both sides of the symmetry axis considered; the number of normal setae on tarsi given as a range due to the variety of observed states at high consistency in the number of specialized setae

^b^Segments on both sides of the symmetry axis considered

^c^Segments with an illegible number of setae excluded

^d^Calculated in relation to the number of cases with a known chaetotactic state on both sides of the symmetry axis

^e^The variable states related to normal setae were treated together

^f^Lower variability range (20–22n) observed for larvae from locality V

Twenty-three out of 45 analyzed metric characters of larvae differed significantly between three populations (I, IV, V; Table 3). The Dunn tests showed differences between each two groups with respect to 21 traits (for the length of setae 1a and Ti II no significant differences between populations were confirmed). Fifteen characters differed between populations I and V, 11 between IV and V, and only three between I and IV (Table 4).

**Table 3:** Statistical significance of morphological variation between populations I, IV, V (results of Kruskal-Wallis test)

Item no.^a^	Character	*P*
1	LB	< 0.001
2	WB	< 0.001
3	PaTr	0.24
4	PaFe	0.27
5	PaGe	0.0057
6	PaTi	0.63
7	PaTa	0.063
8	Odo	< 0.001
9	ASens	0.0022
10	SBa	0.0001
11	PSens	0.027
12	SBp	0.11
13	ISD	0.0001
14	DS	0.78
15	1a (St I)	0.038
16	2a (St II)	0.0054
17	Co I	< 0.001
18	Co II	0.026
19	Co III	0.0053
20	Cx I	0.0030
21	Tr I	0.21
22	bFe I	0.86
23	tFe I	0.068
24	Ge I	0.085
25	Ti I	0.041
26	Ta I (L)	0.052
27	Ta I (W)	0.077
28	Leg I (Cx – Ta)	0.054
29	Cx II	0.16
30	Tr II	0.25
31	bFe II	0.48
32	tFe II	0.088
33	Ge II	0.036
34	Ti II	0.033
35	Ta II	0.063
36	Leg II (Cx – Ta)	0.0096
37	Cx III	0.35
38	Tr III	0.0001
39	bFe III	0.17
40	tFe III	0.49
41	Ge III	0.0034
42	Ti III	0.030
43	Ta III	0.010
44	leg III (Cx – Ta)	0.073
45	IP	0.030

^a^Consecutive traits considered in the analysis

**Table 4:** Morphometric traits revealing statistically significant differences between three populations (α = 0.05)

Character	I : IV	I : V	IV : V
LB		***	***
WB		***	***
PaGe			**
Odo		**	***
ASens			**
SBa	**		***
PSens	*		
ISD			***
1a (St I)			
2a (St II)		**	
Co I		**	***
Co II			*
Co III	**	*	
Cx I		**	*
Ti I		*	
Ge II		*	
Ti II			
Leg II		*	
Tr III		*	***
Ge III		**	
Ti III		*	
Ta III		*	
IP		*	

Dunn’s test of multiple comparisons using rank sum: *0.01 < P < 0.05, **0.001 < p < 0.01, ***P < 0.001

**Fig. 1:**
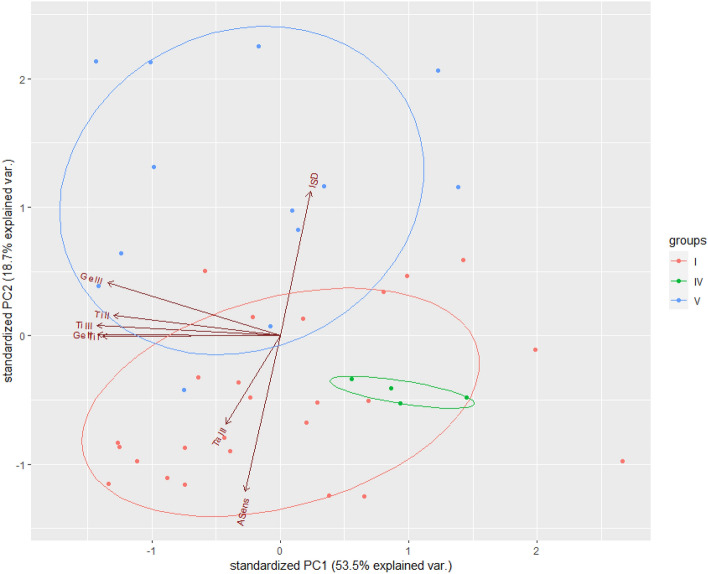
Principal Component Analysis (PCA) results of the morphological variability (based on eight traits) between larvae of *Balaustium murorum* from three populations (I, IV, and V). Individuals plotted against their values for the two first principal components. Dots denote specimens; colors denote the place of origin (groups/localities)

PCA was finally applied for eight variables: ISD, ASens, Ti I, Ge II, Ti II, Ge III, Ti III, and Ta III. The first two principal components explained together 72.2% of the total variation, with the first component (PCA 1) explaining 53.5% and the second (PCA 2) 18.7% of the variation (Fig. 1). When plotted against their respective values for PC1 and PC2 (Fig. 1), population V was shifted from populations I and IV mainly along PC2, indicating more pronounced differences between laboratory-reared larvae (populations I, IV) and field-born ones (population V).

### Modified diagnosis of *Balaustium murorum*

The overall variability range of some morphological traits (number of solenidia on Ti I, Ge II, number of normal setae on Ge II, Ti II, Ge III) observed in specimens examined during the present survey goes beyond the hitherto known for the species and contributes to the modified diagnosis of *B. murorum* in scope pertaining to the larvae. In the updated diagnosis, we consider those character states that have been observed in most specimens, field-born and laboratory-reared ones, on both sides of the symmetry axis (≥ 66%, see also Table 2). The modified diagnosis is as follows: one seta on PaFe; two setae on PaGe; IP < 1200; fn bFe: 4-4-2; fn tFe: 5-5-5; fn Ge: 8-[7–8]-[8–9]; fn Ti: [10–12]-[9–11]-[10–11]; Ti III < 100; 2–3 solenidia on Ti I, 0–1 solenidia on Ge II.

### Comparison

Considering characters whose states exhibit high stability in the analyzed populations, *B. murorum* belongs to the group of species with one seta on palpfemur, fn bFe I-III: 4-4-2 and fn tFe I-III: 5-5-5. Other species that share this character state and have been known from the Palaearctic are *Balaustium kacperi* Haitlinger, *B. nikae* Haitlinger, *B. rajmundi* Haitlinger, *B. wratislaviensis* Haitlinger, *B. biljanae* (Haitlinger), *B. medardi* Haitlinger, *B. minodorae* Haitlinger, *B. soydani* Haitlinger, *B. innocentae* Haitlinger, *B. zhangi* Saboori, *B. akramii* Noei, and *B. ryszardi* Šundić et Noei (Haitlinger 1996, 2000a, b, 2006; Saboori 2001; Noei et al. 2017; Šundić and Noei 2021). A summary of data on leg chaetotaxy of species described/recorded from Poland based on larvae is presented in Table 5. Further comparison – beyond the scope of the present paper – should be backed up with the extensive morphological and molecular studies of the material collected from various localities in the Palaearctic region.

**Table 5:** Comparison of leg chaetotaxy in larvae of *Balaustium* spp. recorded from Poland

Character	*B. murorum*	*B. kacperi*^a^	*B. nikae*^a^	*B. rajmundi*^a^	*B. wratislaviensis*^a^
	Mąkol 2010^a^ and present data^b^	Haitlinger 1996 (n = 1)	Haitlinger 1996 (n = 1), Šundić 2014 (n = 26)	Haitlinger 1996 (n = 1)	Haitlinger 1996 (n = 1)
PaFe	1n	1n	1n	1n	1n
PaGe	2n	2n	2n	2n	2n
Cx I	1n	1n	1n	1n	1n
Tr I	3n	3n	3n	3n	3n
bFe I	4n	4n	4n	4n	4n
tFe I	5n	5n	5n	5n	5n
Ge I	8n, 1σ, 1κ	7n, 1σ, 1κ	7–9n, 1σ	8n, 1σ	7n, 1σ, 1κ
Ti I	10–12n, 2–3ϕ, 1κ	12n, 2ϕ^c^, 1κ	10–12n, 2ϕ^d^, 1κ	12n, 2ϕ, 1κ	11n, 2ϕ^c^, 1κ
Ta I	17–21n, 1ω, 2ζ, 1z	22n, 1ω	18–20n, 1ω, 2ζ^e^, 1z	19n, 1ω	19n, 1ω
Cx II	1n	1n	1n	1n (2n^f^)	1n
Tr II	3n	3n	3n	3n	3n
bFe II	4n	4n	4n	4n	4n
tFe II	5n	5n	5n	4n	5n
Ge II	7–8n, 0–1σ, 1κ	7n, 1σ, 1κ	7–10n^g^, 1κ	8n, 1κ	7n, 1κ
Ti II	9–11n, 2–3ϕ	12n, 2ϕ	11–12n, 2ϕ	12n, 1ϕ, 1κ	10n, 1ϕ
Ta II	(15)17–22n, 1ω, 2ζ, 1z	19n, 1ω, 1z	14–18n, 1ω, 2ζ, 1z	19n, 1ω, 1z	19n, 1ω
Cx III	1n	1n	1n	1n	1n
Tr III	2n	2n	2n^h^	2n	2n
bFe III	2n	2n	2n	2n	2n
tFe III	5n	5n	5n	5n	5n
Ge III	8-9n	8n, 1σ	7–8n	8n	7n
Ti III	10–11n, 1(2)ϕ	11n, 1ϕ	11–12n, 1ϕ	10n, 1ϕ	11n, 1ϕ
Ta III	18–21n, 1ζ	21n	14–18n, 1ζ	17n	19n

^a^Not assigned to L or R

^b^Only the states confirmed on both sides of symmetry axis considered; for frequency of occurrence of particular states see Table 2

^c^Šundić and Noei (2021) assigned the species to the group having two solenidia on Ti I

^d^Two solenidia, instead of one reported by Haitlinger (1996), were confirmed by (Mąkol 2010) during the examination of the holotype

^e^Two eupathidia, instead of none reported by Haitlinger (1996), were confirmed by (Mąkol 2010) during the examination of the holotype

^f^Two coxalae II on one side of symmetry axis reported by Haitlinger (1996)

^g^May also include solenidion

^h^Two setae, instead of three reported by Haitlinger (1996), were confirmed by (Mąkol 2010) during the examination of the holotype

## Discussion

The PCA did not contradict the common species identity of specimens representing all examined populations; however, it visualized the wider than hitherto recognized variability of *B. murorum* over only part of the area of the geographic distribution of the species. It is noteworthy that the differences observed in the field-born and laboratory-reared larvae could be determined by different factors. In the case of field-born specimens, the genetic diversity at the intra-population level should be considered as the primary cause of variation, whereas in the case of laboratory-reared larvae, the epigenetic differences can come to the fore. The discovery of clusters as a result of PCA could potentially point to the presence of distinct species. The latter hypothesis, however, was rejected due to the finding of only one haplotype, at intra- and inter-population sampling. A selection of individuals representing different developmental stages for molecular analyses further confirmed the conspecificity of larvae and active postlarval forms.

In studies carried out by Kayastha et al. (2023) on parthenogenetic tardigrade species, despite the higher haplotype diversity revealed in the case of COI than in ITS-2 marker, sometimes the same COI haplotype was shared between populations from very distinct localities. The identity or close similarity (99.8–100%) between our sequences and those obtained by Hiruta et al. (2018) from Austria and Japan confirmed the wide distribution range of *B. murorum*, as already suggested by Hiruta et al. (2018). Further studies, based on more extensive material from the eastern Palaearctic may further contribute to widening the knowledge of the intraspecific variation of the species in question.

The scope of intraspecific variation of morphological traits discovered for *B. murorum*, wider than hitherto recognized, sheds light on criteria adopted for the description of *Balaustium* spp. and calls for re-assessment of the status of several species, with special reference to those described based on very scarce material. The latter should be also associated with re-examination of the type material of some species assigned to the genus, and verification of the character states that depart from that already known for *B. murorum*. Seven species of *Balaustium* – *B*. *kacperi*, *B*. *murorum*, *B*. *nikae*, *B*. *rajmundi* Haitlinger, *B*. *unidentatum* (Trägårdh), *B*. *wratislaviensis* and *B*. *xerothermicum* Gabryś – have been recorded from Poland to date. For only five of them (*B. kacperi*, *B. murorum*, *B. nikae*, *B. rajmundi*, *B. wratislaviensis*) the larva has been described (Haitlinger 1996; Mąkol and Wohltmann 2012; see also Table 5). Given unrecognized variation of most species of *Balaustium* hitherto described from Poland and some other localities within the Palaearctic, the ultimate assessment of their status is difficult to ascertain at present. It cannot be excluded, however, that at least some of these species share a common identity with *B. murorum*.

The morphological survey of *B. murorum* larvae revealed that some characters are especially prone to variation. This applies mainly to the meristic traits of genua and tibiae of all legs and calls for special caution in adopting these characters in species diagnosis based on limited material. In view of the unstable criteria of species recognition, the detailed re-approval of the usefulness of morphological characters, backed up with molecular identification should be carried out to verify the status of hitherto recognized nominal species.

The latter is especially crucial in view of several factors that drive the variation. The biology and ecology of the species, with special reference to the phenology of a given instar, seem to have a significant impact on the variability of the morphological characters at the intraspecific level. Much greater variability of the larval stages is to be expected in species that hibernate in the egg stage (e.g., Mąkol 2000), due to the duration of this stage and often unfavorable thermal conditions, compared to larvae that emerge from eggs that undergo relatively short incubation during vegetation season. The unfavorable impact of environmental factors cannot be excluded also in the case of species with diapausing eggs. Mąkol (2010) obtained larvae from eggs kept at ambient temperature; however, the low sample size was not sufficient for broader conclusions. Although the lack of ambient temperature control during the present study made it impossible to definitively confirm whether the eggs of *B. murorum* are subject to obligatory hibernation, this is the first time that the hypothesis of diapausing eggs as hibernating instar in *B. murorum* could be accepted by laboratory rearing. Our observations of the natural populations of *B. murorum* indicated also that the appearance of ovigerous females in the late spring was followed by the total absence of active instars already at the beginning of July which corresponds to the oviposition and onset of the diapausing phase.

A higher extent of variability of metric traits (value of CV) in the case of field-born larvae compared to laboratory-reared ones can be explained by a higher number of females that gave origin to larvae randomly collected in a natural environment, but also – in the case of weakly sclerotized structures – by the age-related, different level of engorgement of specimens. This is particularly noticeable in the case of the higher value of basic body measurements in field-collected larvae, which displayed more advanced growth as a consequence of earlier food intake.

A higher than hitherto recognized variation of meristic traits in larvae *B. murorum*, except for the increased sample size examined during the present study and the egg incubation period, should be also attributed to hitherto not studied influence of incubation conditions on the occurrence of developmental anomalies. The assumption for this hypothesis is the lower share (%) of specimens with an equal state confirmed on both sides of the symmetry axis as observed among laboratory-reared larvae. The same chaetotactic formula on the right and left side of the symmetry axis, still optional to the most common mode of distribution of normal and specialized setae, observed on tibiae I, II, III and genua II, III (see also Table 2), in laboratory-reared and in field-born larvae, votes for high cautiousness in applying these characters in species discrimination. The various frequency of distribution of states departing from most commonly observed ones, at still possible asymmetrical states that may be attributed to developmental anomalies, also suggest the need to check the states on both sides of the symmetry axis, on the largest possible number of specimens.

The variation discovered in the homogeneous material, much higher than reported until present at the intraspecific level, not only for the genus accommodated in Balaustiinae, but also for other terrestrial parasitengone mites, once again, argues for the need to analyze larger series of individuals as the basis for the description of new species.

## Conclusions

The integrative studies on larvae of *Balaustium* originating from populations in southwestern Poland confirmed their common species affiliation and identity with *B. murorum*. The status of *Balaustium* species known as larvae and distinguished based on the number of setae, with special reference to genua and tibiae I-III should be verified due to the much wider intraspecific variation of meristic traits pertaining mostly to leg chaetotaxy, as revealed during the present study. The hitherto keys to the identification of *Balaustium* spp., and of Balaustiinae known from larvae should be re-built due to several misinterpreted character states that may result in false identification. In species diagnosis, in the pursuit of recognition of variability at the intraspecific level, it is useful to check the character states on both sides of the symmetry axis due to the discrepancies confirmed during this study. The application of the molecular traits to revise the significance of the diagnostic features commonly adopted in morphology-based species diagnosis is recommended.

## Data Availability

The datasets (the raw measurements) analysed during the current study are available from the corresponding author on reasonable request.
